# Denial of access to sexual and reproductive health services in Europe: an analysis of cases from the European court of human rights (1976–2023)

**DOI:** 10.1186/s12978-025-02247-z

**Published:** 2026-01-09

**Authors:** Elisa Groff, Florian Steger

**Affiliations:** Institute of the History, Philosophy and Ethics of Medicine, Ulm University Hospital, Ulm, Germany

**Keywords:** Access to sexual and reproductive health, Sexual rights, Abortion, Reproductive coercion, Reproductive justice, Medical ethics

## Abstract

In June 2021, the European Parliament agreed to ensure universal and adequate access to high-quality sexual and reproductive health (SRH) care and to promote SRH education in all European Member States, in accordance with the European Convention on Human Rights. The aim of this research was to systematically pursue a qualitative analysis of applications concerning the denial of access to SRH services which have been brought before the *European Court of Human Rights* (ECtHR) up to December 2023 from an ethical perspective. A systematic search of judgments by the ECtHR was conducted in the HUDOC database using a combination of keywords resulting in nine thematic search categories. A thematic analysis approach was then used to describe and interpret the judgments through the lens of intersectionality and according to the four bioethical principles of “autonomy”, “beneficence”, “non-maleficence” and “justice”. *N* = 23 judgments from *n* = 15 EU Member States of the Council of Europe that met the research objectives were included in the descriptive analysis. These confirmed the violation of Articles 3, 5, 8 and 10 of the Convention. Violations of SRH services are indicators of social and public health policies that increase the vulnerability of marginalised groups. Our analysis shows that the discourse on access to SRH needs to shift from reproductive choices being unavailable to marginalised people to reproductive rights. Given the complexity of scientific advances in biomedicine and in response to evolving reproductive health needs, SRH services in Europe need to be monitored by the EU to secure equitable and non-discriminatory access.

## Introduction

On June 24th 2021, the European Parliament declared that “the right to health, in particular sexual and reproductive health rights, is a fundamental pillar of women’s rights and gender equality”. The resolution was passed to improve the situation of sexual and reproductive health and rights in the EU, in the frame of women’s health (2020/2215(INI): https://www.europarl.europa.eu/doceo/document/TA-9-2021-0314_EN.html). With 378 votes in favour, 255 against and 42 abstentions, the plenary called on all “States parties” to the European Convention for the Protection of Human Rights and Fundamental Freedoms (Convention) to ensure universal and adequate access to high quality sexual and reproductive health care (SRH) and to promote SRH education. Therefore, this research analysed 183 applications filed with the *European Court of Human Rights* (ECtHR) under Articles 34 or 35 of the Convention from 1976 to 2023, concerning violations or neglect of SRH rights and services in the EU, Russia, Turkey and the UK [1]. In particular, it critically reviewed (1) the year and circumstances of each case, (2) the country in which it was originated, (3) what articles of the Convention were allegedly violated and (4) the Court’s assessment.

### The European court of human rights

The *European Court of Human Rights* (ECtHR) is an international court established in 1959 to hear applications from individuals or “States Parties” as members of the *Council of Europe*. Under Article 19 of the European Convention on Human Rights (European Court of Human Rights, Council of Europe, 1950/1953), individuals can apply directly to the ECHR to complain about violations of their civil and political rights in their home country, after having failed in their domestic courts (https://fra.europa.eu/en/eu-charter/article/19-protection-event-removal-expulsion-or-extradition; https://www.echr.coe.int/documents/d/echr/50questions_eng) [2–7]. In order to lodge an application in accordance with Article 34, an individual must prove that they were directly affected by the measure complained of. According to Article 35 § 1 of the Convention, the Court can only deal with a case after all domestic remedies have been exhausted.

### Research aims

This research was an ethical analysis of cases brought before the ECtHR up to December 2023 through the lens of intersectionality and according to Beauchamp and Childress’ principles of biomedical ethics. It had three main objectives: (1) to evaluate the determinants of SRH, i.e. availability of resources, accessibility without discrimination, cultural acceptability, quality of facilities and technological innovation ([8]:4–5; [9, 10]); (2) to examine the extent to which the access to SRH services and education is de facto guaranteed within the European Union; and (3) to assess the diversity of European citizens’ experiences of control over their own reproductive choices in the EU. We focused on the content of SRH and rights for women and girls as addressed in the EU Parliament Resolution of 24 June 2021, such as access to legal and safe abortion, comprehensive sexuality education, fertility treatment, perinatal care for mother and baby, provision of SRH services ((2020/2215(INI): https://www.europarl.europa.eu/doceo/document/TA-9-2021-0314_EN.html). In particular, we looked at cases of restriction to and denial of European citizens’ right to informed reproductive self-determination and their right to autonomy and privacy through the lens of reproductive justice [11, 12], and intersectionality in terms of experiencing multiple discriminations simultaneously [13]. This means that we applied an intersectional and holistic reproductive justice framework ([14]:65,69,82,85), which acknowledges the link between socioeconomic status, level of education, gender and race in women’s and transgender and gender diverse individuals’ decisions to have a child, to not have a child, or to parent one in a safe, healthy and sustainable environment ([14]: 9, 16, 54–57; [8, 9, 12]: 17–18). Finally, we discussed the extent to which the Court’s judgments were consistent with the four bioethical principles of “autonomy”, “beneficence”, “non-maleficence” and “justice” ([8, 15]:10–11; [9]). This ethical analysis allowed us to (1) account for the historical and socio-cultural factors that limited the ability of European citizens to make informed and consensual decisions about their reproductive health between 1976 and 2023 ([14]:14); (2) examine the evolution of policies for reproductive rights and SRH over the last forty years in the EU, with a particular focus on the period from 2004 to 2023; and (3) monitor the status of the policies implemented for universal access to SRH care in different countries within Europe ([14]:9]). Finally, the results of the analysis helped us to contextualise the judgments and decision from the European Court in response to current public health and social challenges, thereby broadening our understanding of reproductive ethics in the European context.

## Methods

Judgments by the ECtHR were accessed via the HUDOC database. The first preliminary advance search was conducted on 18th October, 2022 and repeated on 25th July and 31 st December 2023, using the combination of search terms in Table 1. This resulted in 9 categories that encompassed the scope of rights and lack thereof associated with access to SRH.

**Table 1:** Search terms and thematic categories *1–9* used for advanced searching in the HUDOC database: online access on 18th October, 2022 and 25th July and 31 st December 2023

*	First Word	AND	Second Word	AND	Third Word	AND	Fourth Word	AND	Fifth Word	AND	Sixth Word
1	Access		Sexual		Reproductive		Health		Care		
2	Denial		Access		Sexual		Reproductive		Health		Care
3	Abuse		Sexual		Reproductive		Health		Care		
4	Access		Health		Abortion						
5	Access		Health		Reproductive		Coercion				
6	Access		Health		Reproductive		Abuse				
7	Access		Health		Reproductive		Violence				
8	Access		Health		Obstetric		Violence				
9	Access		Health		Contraception						

The search returned *n* = 183 results; of these *n* = 129 were doubles. *N* = 54 were screened. *N* = 23 were included in the descriptive analysis. We selected judgements of Grand Chamber and Chamber.

### Descriptive statistics

Descriptive statistics were conducted on the Articles of the Convention for the Protection of Human Rights and Fundamental Freedoms and of the Protocols, which were referred to in the *n* = 23 judgments. For the sake of clarity, we counted a violation of an article if the Court found at least one element or a subparagraph of the article to be in breach [2–7]. This means that if the court held that there was a substantive violation of an article but not a procedural violation of the same article, or vice versa, we counted as a violation in our analysis. An example of this is the case of V.C. v. Slovakia (18968/07, 2011) concerning the violation of Article 3 (Table 3).

### Reflexive thematic analysis

A reflexive thematic analysis approach was used to describe and interpret the 23 judgments by the ECtHR that we screened as relevant [16]. In the first stage, the content of the judgments was comprehensively read by the first author, organised for analysis in a spreadsheet by year of application, circumstances, articles allegedly violated and the Court’s assessment, and listed under one of the nine thematic search categories resulting from the different combinations of the six keywords in the advanced search of the HUDOC database (Table 1). For judgments falling under more than one category at time, all categories involved were displayed (Table 2).

The nine thematic search categories reflected the possibilities of denial or abuse of SRH care, sexual rights and education, as addressed in the policy approved by the European Parliament in June 2021. In the second stage of the thematic analysis, the content of the judgments was (1) examined for recurring themes, (2) decoded under a conceptual category, (3) analysed in depth, and (4) discussed reflectively within the team of authors in relation to the articles of the Convention, a comparative view of relevant national and international law, and the positive and negative obligations of the Member States of the Council of Europe towards their citizens in matters of access to SRH care and education (Tables 2, 3 and 4).

## Results

**Fig. 1:**
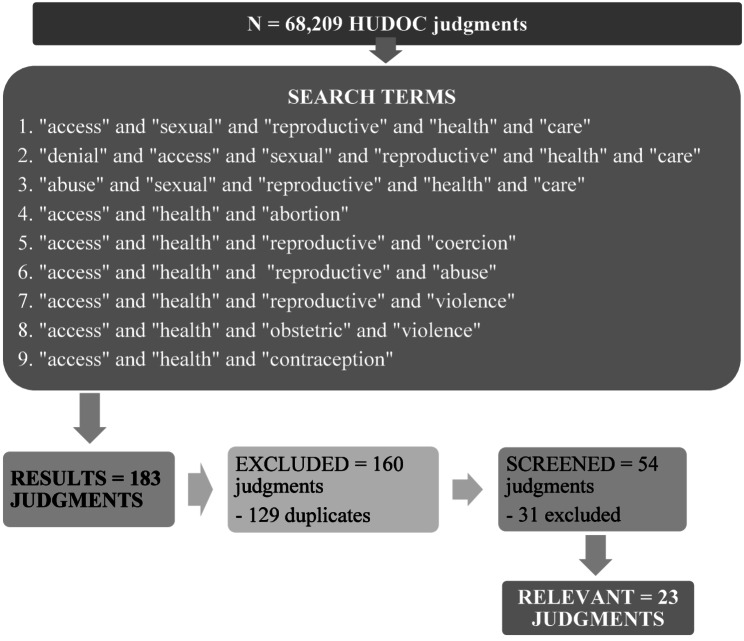
Flowchart of the advanced search with results

Out of the *n* = 68,209 judgments available online on the HUDOC database, our search returned *n* = 183 results, of which *n* = 129 were duplicates. We screened *n* = 54 judgments and included *n* = 23 in the descriptive analysis (Fig. 1):

All judgments were subject to publication when final in accordance with Article 44 of the Convention. We selected judgements of Grand Chamber and Chamber. The search by category yielded the following results (Table 2):

**Table 2:** Search terms and thematic categories *1–9* used for advanced searching in the HUDOC database with results, duplicates and relevant judgments: online access on 18th October, 2022, 25th July and 31 st December 2023

*	1st Word	AND	2nd Word	AND	3rd Word	AND	4th Word	AND	5th Word	AND	6th Word	Results Tot *N* = 183	Dupli-cates *N* = 129	Relevant *N* = 23
1	Access		Sexual		Reproductive		Health		Care			30		14
2	Denial		Access		Sexual		Reproductive		Health		Care	14	12	5
3	Abuse		Sexual		Reproductive		Health		Care			16	16	7
4	Access		Health		Abortion							52	45	4
5	Access		Health		Reproductive		Coercion					8	8	4
6	Access		Health		Reproductive		Abuse					22	15	11
7	Access		Health		Reproductive		Violence					23	21	9
8	Access		Health		Obstetric		Violence					4	4	2
9	Access		Health		Contraception							14	8	9

### Countries

Applications were lodged with the Court against *n* = 25 State Members of the Council of Europe (CoE), of which only 6 were classified by the World Bank as non-high-income countries in 2023, i.e. Albania, Bulgaria, Georgia, Republic of Moldova, Russia and Turkey. On March 16th 2022 the Russian Federation was expelled from the CoE. It is therefore no longer a party to the ECHR. However, we included the 7 judgments resulting from our search in this analysis as they took place in the years 2016 (*n* = 2), 2017 (*n* = 2), 2019 (*n* = 1), 2022 (*n* = 1) and 2023 (*n* = 1). We also screened the last case of January 2023 as it was originated in three applications regarding the Russian Federation in July 2010, April and May 2014. The *n* = 23 applications included in our descriptive analysis were lodged with the Court under Articles 34 or 35 of the Convention for the Protection of Human Rights and Fundamental Freedoms against *n* = 15 State Members: Austria, Croatia, Czech Republic, Denmark, France, Ireland, Italy, Latvia, Lithuania, Poland, Russia, Slovakia, Sweden, Turkey, UK (Table 3).

**Table 3:** Member States of the Council of EU against which applications were lodged with the ECtHR, year of the application, no. of relevant applications (x = not relevant), no. of applications included in the descriptive analysis

	Member States of the Council of EU	*N* = applications 1976–2023	Year of application	Relevant *N* = 23	Not Relevant
1	Albania	1	2020		X
2	Austria	2	2010/2011	1	1
3	Bulgaria	1	2021		X
4	Croatia	1	2019	1	
5	Czech Republic	4	2014/2016/2021/2022	2	2
6	Denmark	2	1976/2022	1	1
7	France	4	2003/2004/2012/2017	1	3
8	Germany	1	2020		X
9	Georgia	1	2022		X
10	Ireland	2	1992/2010	2	
11	Italy	3	2012/2015/2012/	2	1
12	Latvia	1	2014	1	
13	Lithuania	2	2018/2023	1	1
14	Norway	1	2022		X
15	Poland	4	2007/2011/2012/2013	3	1
16	Portugal	2	2012/2017		X
17	Republic of Moldova	1	2022		X
18	Romania	2	2017/2020		X
19	Russia	7	2016/2016/2017/2017/2019/2022/2023	3	4
20	Slovakia	2	2009/2011(2012)	2	
21	Spain	1	2014(2012)		X
22	Sweden	1	2012	1	
23	Switzerland	2	2011/2012		X
24	Turkey	1	2015	1	
25	UK	2	1976/2013	1	1

### Articles of the European convention on human rights

Significantly, 73,9% of the applications concerned an alleged violation of Article 8 of the Convention, which was upheld in *n* = 10 of the judgments. 30,4% concerned an alleged violation of Article 14, which was confirmed in *n* = 1 judgment. 26,1% concerned an alleged violation of Articles 3 and 13. A violation of Article 3 was confirmed in *n* = 3 judgments. With regard to Article 13, the Court did not consider it necessary to examine the Article separately. 17,39% of the applications concerned an alleged violation of Article 10, which was confirmed in *n* = 3 judgments (Table 4).

**Table 4:** Frequency and no. of applications dealing with alleged violations and confirmed violations of an Article of the convention including sub-paragraphs

Articles of the European Convention on Human Rights	Alleged Violations	Violations	Countries/year
2: the right to life	*N* = 2; 8,6%	*N* = 0	
3: freedom from torture and degrading or inhuman treatment	*N* = 6; 26,1%	*N* = 3	Poland 2011, 2012 Slovakia 2011
5: the right to liberty and security	*N* = 1; 4,34%	*N* = 1	Poland 2012
6: the right of access to a court	*N* = 2; 8,6%	*N* = 1	Slovakia 2009
7: no punishment without law	*N* = 1; 4,34%	*N* = 0	
8: the right to respect for private and family life, home and correspondence	*N* = 17; 73,9%	*N* = 10	Ireland 2010 Italy 2013 Latvia 2014 Poland 2007, 2011, 2012 Slovakia 2009, 2011 Russia 2016, 2022
9: freedom of thought, belief and religion	*N* = 1; 4,34%	*N* = 0	
10: the right to freedom of expression	*N* = 4; 17,39%	*N* = 3	Ireland 1992 Lithuania 2023 Russia 2017
12: the right to marry and to found a family	*N* = 1; 4,34%	*N* = 0	
13: the right to have an effective remedy before a national authority	*N* = 6; 26,1%	*N* = 0	
14: protection from discrimination in respect of the Convention’s rights and freedoms	*N* = 7; 30,4%	*N* = 1	Russia 2017
18: limitations on use of restrictions of rights	*N* = 1; 4,34%	*N* = 0	

### Thematic search categories

Of the *n* = 23 applications included in the descriptive analysis, duplications occurred when an application crossed categories and therefore fell into different categories more than once (Table 2). Table 5 below shows the details of each application included in the descriptive analysis, with information on the year, the country against which it was lodged in alphabetical order, and the thematic search categories involved.

**Table 5:** No. of applications included in the descriptive analysis with information about the year and the country in which they were lodged, and details of the violations relating to access to sexual and reproductive health care (SRHC) according to the thematic search categories 1–9

	A = Applications *n* = 23	Thematic Search Categories 1–9. SRHC = sexual and reproductive health care. AtH = access to health.								
	Case/Country/No./Year	Access to SRHC *N* = 14/23	Denial of Access to SRHC *N* = 5/23	Abuse of SRHNN = 7/23	AtH and Abortion *N* = 4/23	AtH and Repro-ductive Coercion *N* = 4/23	AtH and Repro- ductive Abuse *N* = 11/23	AtH and Repro- ductive Violence *N* = 10/23	AtH and Obstetric Violence *N* = 2/23	AtH and Contra-ception *N* = 9/23
A1	S.H. and Others v. Austria, 57,813/00, 2011	X	X	X			X			
A2	Pojatina v. Croatia, 18,568/12, 2018						X			
A3	Dubská and Krejzová v. Czech Republic, 28,859/11 and 28,473/12, 2014	X						X	X	
A4	Pejrilova v. Czech Republic, 14,889/19, 2023	X								X
A5	Kjeldsen, Busk Madsen and Pedersen v. Denmark, 5095/71, 5920/72, 5926/72, 1976	X		X			X			X
A6	V.O. v. France, 53,924/00, 2004							X		X
A7	Open Door and Dublin Well Woman v. Ireland, 14,234/88; 14,235/88, 1992		X							
A8	A, B,C v. Ireland, 25,579/05, 2010	X								X
A9	Costa and Pavan, v. Italy, 54,270/10, 2013	X								
A10	Parrillo v. Italy, 46,470/11, 2015						X			
A11	A.K. v. Latvia, 33,011/08, 2014				X			X		
A12	Macatè v. Lithuania, 61,435/19, 2023	X		X		X	X	X		X
A13	Tysiac v. Poland, 5410/03, 2007	X								
A14	R.R. v. Poland, 27,617/04, 2011	X	X	X	X					
A15	P. and S. v. Poland, 57,375/08, 2012	X	X	X	X	X	X	X		X
A16	Y.Y. v. Russia, 40,378/06, 2016				X					
A17	Bayev and Others v. Russia, 67,667/09, 2017	X	X	X		X	X	X		
A18	Y.P. v. Russia, 43,399/13, 2022	X		X			X	X		X
A19	K.H. and others v. Slovakia, 32,881/04, 2009						X	X		
A20	V.C. v. Slovakia, 18,968/07, 2011	X				X	X	X	X	X
A21	Vejdeland and Others v. Sweden, 1813/07, 2012						X	X		
A22	Y.Y. v. Turkey, 14,793/08, 2015	X								
A23	Handyside v. UK, 5493/72, 1976									X

#### Access to sexual and reproductive health care

*N* = 14 applications (Table 5) concerned different aspects of “access to sexual and reproductive health care”. The case law ranged from denial of legal abortion (A15, A14, A8) or therapeutic termination of pregnancy (A13), to forced sterilisation (A20, A18), denial of home birth (A3), prohibition of the use of a deceased partner’s sperm (A4), denial of access to preimplantation genetic diagnosis (A9), denial of heterologous artificial procreation techniques (A1), denial of gender reassignment surgery (A22), and prohibition of printed material and dissemination of information on sex education and diversity (A12, A5, A17).

#### Denial of access to sexual and reproductive health care

*N* = 5 applications (see Table 5) concerned the alleged “denial of access to” legal termination of pregnancy (A15, A14), heterologous assisted procreation (A1), non-directive counselling (A7), and freedom of speech against sexual discrimination (A17).

#### Abuse of sexual and reproductive health care

*N* = 7 applications (Table 5) concerned the alleged “abuse” in various areas related to “sexual and reproductive health care”, e.g. prohibition of legal abortion (A15, 2), prohibition of heterologous assisted reproduction (A1), restrictions on the right to freedom of expression in matters of sexual diversity and education (A12, A5, A17), and forced sterilisation (A18).

#### Access to health and abortion

*N* = 4 applications (Table 5) dealt with allegations against the right of access to legal abortion (A15, A14, A11, A16).

#### Access to health and reproductive coercion

*N* = 4 applications (Table 5) concerned coercive behaviours infringing on the applicants’ reproductive and human rights, e.g. denial of legal abortion (A15), sterilisation without patient consent (A20), restriction of the right to publish on sexual and reproductive diversity (A12), and express public support for homosexual information (A17).

#### Access to health and reproductive abuse

*N* = 11 applications (Table 5) concerned different forms of “abuse” to control the applicants’ rights and choices in relation to their immediate or future reproductive health. The search yielded results on denial of legal abortion (A15), forced sterilisation (A19, A20, A18), denial of embryos donation for research (A10), denial of heterologous assisted procreation (A1), denial of home birth (A2), public debate on sex education (A12, A5, A17, A21).

#### Access to health and reproductive violence

*N* = 10 applications (see Table 5) contained allegations of “reproductive violence” in access to health. These included cases of denial of legal abortion (A15, A11), forced sterilisation (A19, A20, A18), denial of home birth (A3), reproductive surgical ill-treatment (A6), restriction of freedom of expression in matters of sexual content (A12, A17, A21).

#### Access to health and obstetric violence

*N* = 2 applications (Table 5) dealt with allegations of “obstetric violence” in access to health. The cases in question concerned sterilisation without patient consent (A20) and denial of home birth (A3).

#### Access to health and contraception

*N* = 9 applications (Table 5) resulted from the combinations of the search terms “access to health” and “contraception”. These included cases of denial of abortion (A15, A8), sterilisation without patient consent (A20, A18), denial of assisted reproduction (A4), restriction of freedom of expression in matters of sex education (A12, A5, A23), and reproductive surgical ill-treatment (A6).

### Decoding conceptual categories

In the second stage of our analysis, we read the textual content of the *n* = 23 judgments in depth and detail, we identified recurring themes, and we decoded the data into eleven conceptual categories for interpretation and discussion [10, 16]. Table 6 below shows the 11 conceptual categories after screening with references to the number of applications concerned and the country against which the application was lodged.

**Table 6:** Thematic categories after screening, n = of applications, country of application. A = application as highlighted in Table 5

	Decoded Conceptual Categories	*N* = 23 applications	Country of applications
1	Denial of access to legal termination of pregnancy	4 (A8, A13, A14, A15)	Ireland (*n* = 1) Poland (*n* = 3)
2	Denial of access to non-directive counselling	1 (A7)	Ireland
3	Denial of access to prenatal testing	1 (A11)	Latvia
4	Denial of access to assisted reproduction technologies	3 (A4, A9, A1)	Czech Republic (*n* = 1) Italy (*n* = 1) Austria (*n* = 1)
5	Refusal of embryos donation for research	1 (A10)	Italy
6	Coercive sterilisation	3 (A19, A20, A18)	Slovakia (*n* = 2) Russia (*n* = 1)
7	Denial of access to postnatal healthcare	1 (A16)	Russia (*n* = 1)
8	Refusal of home birth	2 (A2, A3)	Czech Republic (*n* = 1) Croatia (*n* = 1)
9	Reproductive ill-treatment	1 (A6)	France
10	Impaired access to sex education and information	5 (A12, A13, A17, A23)	Denmark (*n* = 1) Lithuania (*n* = 1) Russia (*n* = 1) Sweden (*n* = 1) UK (*n* = 1)
11	Denial of access to sexual healthcare	1 (A22)	Turkey

#### Cases of denial of access to legal termination of pregnancy

*N* = 4 judgments concerned the denial of the right to have a lawful termination of pregnancy and access to information on pre- and post-abortion care. Significantly, 3 out of 4 cases arose in Poland, 1 in Ireland. In each case, the Court unanimously found a violation of Article 8, whose main purposes are to secure the effective respect for the privacy and personal autonomy of citizens and to protect them against arbitrary interference by public authorities. In the applications lodged against Poland, doctors failed to secure these essential objects. Their behaviour collided with the National Health Act of 1993, which allows an abortion on medical grounds and when the pregnancy is the result of an illegal act.

In the case of P. and S. v. Poland (57375/08, 2012), the medical professionals negated the applicants the possibility of a lawful abortion, even though the pregnancy had resulted from rape; while in Tysiac (5410/03, 2007) and R.R. v. Poland (27617/04, 2011), the doctors refused to issue a certificate of termination of therapeutic pregnancy, as in the case of A, B, C v. Ireland (25579/05, 2010). Furthermore, in the application of P. and S. v. Poland (57375/08), the Court considered four aggravating factors, such as the removal of the applicant from the custody of her mother, the disclosure of information about her case to the general public, the investigation into allegations of unlawful intercourse, and the harassment the applicants received by pro-life activists, members of the Catholic Church and the press, all of which led to the judgment of additional violations of Articles 5 and 3 of the Convention.

#### Case of denial of access to non-directive counselling

The *n* = 1 case of Open Door and Dublin Well Woman v. Ireland (14234/88; 14235/88, 1992) dealt with the accusation of unlawful counselling about induced abortion against the applicants, who in turn claimed that they had been denied their right - under Article 10 of the Convention- “to hold opinions” and to “impart information” about sexual and reproductive health “without interference by public authority”. The applicants used to offer health services for women that covered the whole spectrum of sexual and reproductive wellbeing, e.g. from pap smear tests to breast examinations, information on contraception methods, IVF and non-directive counselling. The Court confirmed the violation of Article 10.

#### Cases of denial of access to prenatal testing

In the *n* = 1 case of A.K. v. Latvia (33011/08, 2014), the applicant complained of having been denied timely access to prenatal screening, such as an AFP test, to identify the trisomy 21 in her daughter. The Court found a procedural violation of Article 8.

#### Cases of denial of access to assisted reproduction technology

*N* = 3 applications are complaints about state interference with the exercise of the applicants’ rights to have recourse to techniques of artificial procreation. They led to allegations of a violation of Article 8, which the Court upheld only in the case of Costa and Pavan v. Italy (54270/10, 2013). In the case of S.H. and Others v. Austria (57813/00, 2011), the 4 applicants, 2 married heterosexual infertile couples, were denied access to heterologous artificial insemination. This would have been the only means by which one of the partners could be the genetic parent of a child conceived by IVF, using a donor of sperm or oocytes respectively. In the case of Costa and Pavan v. Italy (54270/10, 2013) the applicants complained about a blanket ban on the use of preimplantation genetic diagnosis (PGD). They were denied access to assisted reproduction, and in particular to PGD, because the disease of cystic fibrosis, of which they were healthy carriers, was not listed among the diseases for which access to PGD was permitted under Italian legislation. The case of Pejrilova v. Czech Republic (14889/19, 2023) concerned the applicant’s wish to use her late husband’s cryopreserved sperm posthumously.

#### Cases of refusal of embryos donation for research

In the case of Parrillo v. Italy (46470/11, 2015), the applicant was refused permission to donate her and her late husband’s embryos for research, in violation of Italian Law No. 40/2004. The Court found no breach of Article 8 of the Convention, as alleged by the applicant. It referred to the European Centre for Law and Justice, according to which, in the present case, the interest of science did not outweigh the respect for the embryo in accordance with Article 2 of the Oviedo Convention (1997), i.e. the principle of the “primacy of the human being”.

#### Cases of refusal of home birth

*N* = 2 applications claimed the denial of the possibility of giving birth at home with the assistance of a health professional: case of Pojatina v. Croatia (18568/12, 2018), and cases of Dubská and Krejzová v. Czech Republic (28859/11 and 28473/12, 2014).

Although the Court found that the right to decide on the circumstances of childbirth did fall within the scope of the private life provision of Article 8 of the Convention, it declared that there had been no violation on the part of the authorities. The centrality of the Court’s arguments was the right of the domestic authorities to adopt the available laws on home birth. In sum, both Croatian and Czech legislation did not consider home birth to be a *lege artis* procedure because of the dangers associated with an out-of-hospital setting. As a result, home births were not covered by public health insurance, and midwives registered as state health professionals were not authorised to practise outside hospital, which in turns meant that postnatal care was denied to mother and baby after home delivery. The law was based on the opinion of medical experts.

#### Cases of coercive sterilisation

*N* = 3 cases concerned the practice of sterilisation in public hospitals during caesarean section without the patients’ informed consent and not as a life-saving measure. In all three cases the Court held that there had been a violation of Article 8 of the Convention, as the irreversibility and the coercion of the procedure constituted a major interference with the women’s reproductive choices in terms of their personal autonomy and development.

In the case of Y.P. v. Russia (43399/13, 2022) the applicant was forced to resort to IVF, without medical indication and in disregard of her autonomy. The *n* = 2 applications v. Slovakia concerned women of Roma ethnic origin (K.H. and others v. S., 32881/04, 2009; V.C. v. S., 18968/07, 2011). In addition to being subjected to coercive sterilisation, both applicants were denied the same standard of health care and respect as other patients by being segregated in “gipsy accommodations” during hospitalisation and by not having full access to their medical records. In this respect, the Court found an additional violation of Article 6 of the Convention in K.H. and others v. S. (32881/04), and a substantive violation of Article 3 in V.C. v. S. (18968/07) referring to the degrading treatment she received.

#### Case of denial of access to postnatal healthcare

In the case of Y.Y. v. Russia (40378/06, 2016), the denial of access to adequate postnatal care services was intertwined with a lack of protection against arbitrariness by public authorities, which the Court found to be incompatible with Article 8 of the Convention. Indeed, in response to the applicant’s complaint about the hospital’s failure to provide timely and adequate intensive care for her first twin, which died hours after birth, the Committee for Healthcare at the St Petersburg City collected and examined the applicant’s medical records without her consent. As a result of this unlawful investigation, which was also in violation of Articles 23 and 24 of the Constitution of the Russian Federation, the Committee claimed that the applicant’s premature delivery and the subsequent death of one of her twins were due to urogenital infections and her previous “artificial abortions”.

#### Case of reproductive ill-treatment

The case of V.O. v. France (53924/00, 2004) concerned the death of the applicant’s foetus in hospital, which the mother claimed to be homicide.

#### Cases of impaired access to sex education and information

*N* = 5 applications involved the denial or restriction of children’s access to sex education and information about the sexuality spectrum due to discrimination based on gender identity and sexual orientation.

In the case of Busk Madsen and Pedersen v. Denmark, (5095/71, 5920/72, 5926/72, 1976), the applicants, who were three couples of parents, argued that integrated compulsory sex education, as introduced into State schools by the 1970 Danish Act, was contrary to the beliefs they held as Christian parents and constituted a violation of Danish legislation (Art. 2 of Protocol No. 1), which read that: “the State shall respect the right of parents to ensure such education and teaching in conformity with their own religious and philosophical convictions”.

The case of Macatè v. Lithuania (61435/19, 2023) involved the suspension of the distribution of the applicant’s children’s book for one year, which was later resumed with warning labels for individuals under 14 years of age. The book contained a collection of six fairy tales, two of which depicted same-sex relationships and marriage. The Court found that the removal of the book from sale was unmotivated and violating Article 10 of the Convention. Moreover, the Court added that (1) mandatory sex education in schools was compatible with the Lithuanian legislation (Article 2, Protocol No. 1), as well as with Articles 8 and 9 of the Convention (p.67); and (2) “with respect to the best interests of the child, [.] there was no scientific evidence [.] suggesting that the mere mention of homosexuality, or open public debate about sexual minorities [.], would adversely affect children” (p. 68).

In the case of Bayev and Others v. Russia (67667/09, 2017), the applicants, who were LGBTQ + activists, were found guilty of public homosexual propaganda that was deemed harmful to children’s health and development (Russian Federal Law No.436-F3, 2015, Sects. 5 and 124-FZ, 1998, Sect. 14). The Court determined that the ban on public statement was discriminatory and violated both Article 10 on “freedom of speech” and Article 14 on “the enjoyments of (the Covention’s) rights and freedoms […] without discrimination […]” (cf. the position of the “Venice Commission” on the discrimination of “homosexual propaganda” v. “heterosexual propaganda” in Europe: CDL-AD(2013)022).

The case of Vejdeland and Others v. Sweden (1813/07, 2012) involved the attempted boycott of sex education in Swedish schools. The applicants, who were anti-LGBTQ + activists, argued that Swedish education on sexuality would be detrimental to the health and morals of minors. They claimed that their right to express this concern was violated by the authorities. The Court found that there was no violation of Article 10 of the Convention on “freedom of speech”.

The case of Handyside v. UK (5493/72, 1976) involved the conviction of the applicant for having translated a children’s book for publication, which was deemed obscene and anti-authoritarian under the national Obscene Publications Acts 1959/1964 (Sect. 3). The applicant claimed that he prepared the English version of this Danish book with the help of a group of children and teachers. The review process ensured that the Chapter on “Sex” was appropriate for school-age children, but comprehensive. The book “was meant to be a reference book” to consult for help and advice on sexual matters, such as masturbation, orgasm, contraceptives, menstruation, child-molesters, pornography, impotence, homosexuality and abortion. The Court did not find any violations of Articles 10, 18 or 14. However, the decision was not on the book being deemed obscene or unlawful, but rather out of respect for the national margin of appreciation.

#### Case of denial of access to sexual healthcare

The case of Y.Y. v. Turkey (14793/08, 2015) involved the denial of access to gender reassignment surgery. It concerned the exercise of medical authority over a citizen’s sexual rights and reproductive choices based on stigma. The Turkish authorities justified their refusal of gender reassignment surgery as an action taken in the public interest, with the aim of preventing undesirable imitative behaviour and preserving the applicant’s reproductive capability. The Court considered that these were not “legitimate aims”, compatible with the values of a “democratic society”. It thus found that the long refusal of the authorities was unlawful and that violated Article 8 of the Convention - see also the report on “Discrimination and violence against individuals based on their sexual orientation and gender identity” by the UN High Commissioner for Human Rights, 2015.

## Discussion

We identified eleven subcategories to discuss the *n* = 23 judgments regarding violations or neglect of SRH, as addressed by the European Parliament’s session in June 2021. By applying an intersectionality [13] and rights-based approach [8, 9], we focused on the patient’s ability to make autonomous, informed reproductive choices in the context of European health care. We also assessed the determinants of SRH, such as poverty, gender, age, ethnicity and level of education, which influence a woman’s decision to have children ([8]:4–5; [9]). Therefore, we framed the discussion in terms of providing adequate, affordable, respectful and equal access to quality resources for SRH services and education, by moving beyond the rhetoric of reproductive choice towards reproductive justice [12, 15].

### Legal and safe access to abortion

The judgments concerning the denial of access to a legal and safe termination of pregnancy frame abortion as a matter of human rights ethics [8, 9, 11]. They acknowledge that foetal and maternal health are intimately connected (see also V.O. v. France, 53924/00, 2004). Moreover, our findings show that where religion interferes with the ethical framework protecting healthcare, such as referrals for abortion – Poland and Ireland are among the European countries with the largest shares of Catholics [17] – patients are left to fend for themselves, without adequate information or alternative services to turn to [18, 19]. These religious restrictions on healthcare have a particularly dramatic impact on vulnerable patients, for example women with lower social status or less financial freedom in the context of an unintended pregnancy.

On this basis, the Convention imposes a positive obligation on each signatory State to ensure that the psychological and physical integrity of pregnant women is respected without any discrimination, which includes guaranteeing access to safe and legal abortion. From an ethical perspective, this recognises the moral status of the pregnant woman as a patient and thus focuses on the balance between prima facie beneficence-based obligations guiding clinical judgment vis-à-vis autonomy-based obligations towards the pregnant woman [20]. Contrary to the Irish Abortion Regulations (Art 40.3), neither international law nor comparative standards permit abortion to protect the life of the mother but not her health. According to the principle of international human rights law recognised in the Convention, the life and health of citizens are equally deserving of state protection (see A, B, C v. Ireland 25579/05, 2010). This means that full access to reliable and transparent information about the condition of the foetus and the impact of the pregnancy on the woman’s health is not only a right of the pregnant woman, but also a central element of SRH services. Access to diagnostic services is thus essential for the pregnant woman to be able to make an informed decision on the possibility of abortion. This healthcare code of conduct is consistent with the four constitutional elements of public healthcare facilities set by the Committee on Economic, Social and Cultural Rights (E/C.12/2000/4), i.e. availability, accessibility, acceptability and of high quality [8; 9].

### Non-directive counselling

Non-directive counselling involves “offering but not recommending” clinical interventions [20, 21]. This means that, in the context of an unplanned pregnancy, consulted health professionals have an autonomy-based ethical obligation to offer guidance without imposing a solution [20]. They are therefore obliged to provide the pregnant woman, who is the patient, with all available alternatives, including termination of pregnancy [22].

### Prenatal testing

In the case of A.K. v. Latvia (33011/08, 2014) there are three specific issues at stake: the failure of the gynaecologist to fulfil his positive obligation to inform the applicant of the availability of a prenatal test; the right of the pregnant woman to have access to information concerning the condition of the foetus, which is directly linked to her own health; and the right of the applicant to decide on the continuation of pregnancy. These are similar circumstances to those in R.R. v. Poland (27617/04; see 4.1 above), where the pregnant woman was denied access to diagnostic services in order to prevent her from making an informed decision about the possibility of abortion (see e.g. the case of Costa and Pavan v. Italy in 4.4 below; and the Belgian Assisted Reproduction Technology Act of 6 July 2007). Access to information is a fundamental aspect about the ability to make informed decisions about healthcare, which has been here utterly overlooked.

### Assisted reproduction technology

The judgments concerning assisted reproduction technologies show that the field of gamete donation for IVF is subject to continuous and dynamic scientific developments that raise legal and ethical issues [23]. The ethical considerations pertain mainly the definition of parental responsibility, the anonymity of sperm donors, the limit of sperm donation and its posthumous use, and related rights of children. At the time when the case of S.H. and Others v. Austria (57813/00, 2011) was examined by the Austrian Constitutional Court, gamete donation for IVF was prohibited in Austria under the civil law principle of *mater semper certa est*, and the Artificial Procreation Act allowed only homologous techniques. Since February 2015 egg donation has been legal in Austria, and heterosexual and lesbian couples can use donor sperm for insemination. Similarly, the restrictions of the Czech law in the case of Pejrilova v. Czech Republic (14889/19, 2023) on the use of assisted reproductive techniques (ART) to heterosexual couples and *inter vivos* had at their core the right of the unborn child not to be orphaned. On this basis, the applicant was denied access to ART after the death of her husband because she did not longer form part of an infertile couple [24]. The circumstances of this case resonate strongly with a current debate questioning the complexities and ethical challenges of post-mortem reproduction in fertility preservation for oncology patients [25, 26].

In the case of Costa and Pavan v. Italy (54270/10, 2013), the Court confirmed a violation of Article 8 due to a lack of consistency in the Italian legislation, which would have allowed the abortion of a foetus with cystic fibrosis, but it prohibited PGD to detect the presence of the affected genes. Indeed, cystic fibrosis is an untreatable “genetic disease of particular gravity” and as such is included among the “medical indications” for the “use of PGD” in the document on preimplantation and prenatal genetic testing published by the Steering Committee on Bioethics of the Council of Europe on 22 November 2010 (CDBI/INF [2010] 6; see also the text of the Oviedo Convention, 1997, which was not ratified by the Italian Government) [27]. Significantly, the report “Preimplantation Genetic Diagnosis in Europe” published by the Joint Research Centre of the EU Commission in 2007 (EUR 22764 EN), showed that patients from countries where PGD was prohibited went abroad for the diagnosis, e.g. to Spain, Belgium, the Czech Republic or Slovakia.

To date, there is no established European consensus on IVF treatment and PGD. The use of assisted reproductive technologies in Europe is still regulated by different national laws, professional guidelines, and constitutional principles. The inevitable consequence is that Member States of the CoE have a wide margin of discretion on ethical and social issues, and the implications are alarming for the global fertility tourism, which is booming [28]. However, as citizens’ reproductive health needs evolve, authorities need to strike a fair balance between prudence and the social realities of their citizens (CDBI-CO-GT3 2003) [29, 30].

### Cryopreserved embryo donation

Cryopreserved embryo donation is a timely area of research with expanding potential, particularly relevant for fertility treatments and fertility preservation for oncology patients [24, 26]. In 2000, The European Group on Ethics in Science and New Technologies stated that the ethical aspects of human stem cell research and use fell within the competence of each Member State of the CoE (for an updated overview as of 2023 see https://www.eurostemcell.org/regulation-stem-cell-research-europe). Accordingly, European countries apply different systems of regulation to stem cell research, including laws and professional standards. This testifies to the complexity of the scientific advances and ethical challenges in this evolving field, to which European domestic policies have adapted in the context of their national history, cultures and biopolitics [31, 32]. The moral status attached to embryos and the meaning given to embryo donation as a socio-moral practice, as a form of solidarity with research or with an open future, have called for the questioning of donors’ motivations in qualitative studies [33–35]. This meets a pressing demand from researchers to reach a consensus at European and international level on the ontological and ethical status of surplus stored embryos, in order to overcome the conflicts of interest between science and moral ethics [36].

### Home birth

The judgments concerning the state denial of access to safe home birth reflect the current scientific debate on the subject ([14]:188–190). The demand for home births has increased in high-income countries worldwide over the last decades. Although scepticism about planned home births compared to planned hospital births has been widely documented in the literature ([20]:205–206; [37, 38]), recent studies show that home births can meet evidence-based standards [39] and be a recommended alternative when out-of-hospital services are needed, e.g. during the COVID-19 pandemic [40]. To date, there is no European consensus on this issue.

### Coercive sterilisation

The elements of discrimination reported in the *n* = 3 cases of coercive sterilisation are a good starting point for understanding what makes an individual vulnerable to the reproductive healthcare [41:57–94]. The case of Y.P. v. Russia (43399/13, 2022) is a striking example of medical paternalism, in which the doctor regulated the applicant’s sexuality and reproduction by forcing her to resort to IVF, without medical indication and in disregard of her autonomy. Respect for patient autonomy is the first ethical principle in medical ethics, and as such is also guaranteed by Article 5 of the Convention on Human Rights and Biomedicine. The applications v. Slovakia (K.H. and others v. S., 32881/04, 2009; V.C. v. S., 18968/07, 2011) concerned the segregation of Roma women in hospital rooms separate from standard patients and health services. All this accounts for the historical and socio-cultural factors that have limited the ability of Roma women to make informed and consensual decisions about their reproductive health due to a “double” race- and- gender-based discrimination ([12]:19; [13, 41]:87). Indeed, accounts of the sterilisation of Roma women are documented under the communist regime in Czechoslovakia from the early 1970 s (V.C vs. Slovakia 2011: p.8) and entered in the Criminal Code (Article 246b). Reports by Human Rights Watch and Amnesty International described the unlawful practice as still in vogue in the early 1990s. The issue was showcased in the UN General Recommendations for Action (No. 20) adopted in 1999 by the Committee on the Elimination of Discrimination against Women and by the European Commission against Racism and Intolerance, which published its third report on Slovakia in January 2004 (V.C vs. Slovakia 2011: p.18). In 2005 the Slovak Public Health Act included sections regarding unlawful sterilisation, informed consent and access to medical record.

The judgments on coercive sterilisation reported here clearly show that policies of denying access to the means to make informed and consensual decisions about one’s reproductive health shift the discourse from reproductive choices, which are unavailable to vulnerable and less privileged patients, to reproductive rights.

### Postnatal care

In the case of Y.Y. v. Russia (40378/06), the applicant’s reproductive choices were medicalised and transformed into a morbidity that marked her and her children [42, 43]. The stigmatisation of abortion among the objective findings of the Committee’s medical report on the applicant’s health status demystifies why healthcare is a human rights issue [44]. The conceptualisation of diseases associated with sexual behaviour as a punishment for sin has a long history in medical discourse [45]. In our example, the exercise of medical authority over the applicant’s sexual rights and reproductive choices was used to evade responsibility for the hospital’s actions and to exclude the applicant from legitimate postnatal health services and reproductive justice [10; 11; 22].

### Foeticide

The case of V.O. v. France (53924/00, 2004) raised the ethical question of whether harming a foetus should be treated as a criminal offence in line with Article 2 of the Convention, which reads: “1. Everyone’s right to life shall be protected by law […]”. The Court concluded that there was no violation of Article 2 and that the applicant’s recourse to the administrative courts was an effective remedy. The central question raised by this application – as to whether the definition of “everyone” in Article 2§ 1 should be applied to the foetus – approaches the biomedical debate of the rights of the foetus compared to those of the mother, as discussed in the cases of Tysiac (5410/03, 2007), R.R. (27617/04, 2011) and P. and S. (57375/08, 2012) v. Poland, and A, B,C v. Ireland, (25579/05, 2010). Yet, it does so in the context of an unplanned induced abortion, i.e. foeticide, and from the perspective of the foetus. Nonetheless, the solution to this ethical dilemma remains the same as the one we proposed in Sect. 4.1 above: the focus should be on the patient and on the beneficence-based obligation of the doctor towards his patient, namely the pregnant woman ([20]:205).

This case is also relevant to the present discussion for four additional points: (1) the Oviedo Convention on Human Rights and Biomedicine intentionally refrains from providing a precise definition of the term “everyone” out of respect for national sensitivities; (2) the lack of a European consensus on this matter indicates that the issue is constantly evolving to adapt to the latest advances in biomedicine; (3) the Convention recognises that foetal and maternal health are connected; (4) in the case of V.O. v. France (53924/00), the ill-treatment that led to the death of the foetus and the subsequent therapeutic abortion was performed without informed consent due to language barriers. This medical behaviour violated all four biomedical principles of autonomy, beneficence, non-maleficence and justice.

### Sex education

The judgments regarding the provision of sexual information emphasise that children’s education should be pursued for knowledge and not for ideology. In a democratic society, it is necessary to tolerate even unsettling information or ideas “that are opposed to one’s own convictions” (Bayev and Others v. Russia, 67667/09, p. 29), while simultaneously safeguarding the reputation and rights of all citizens. The fact that “some people might find certain types of families or relationships objectionable or immoral” cannot “justify preventing children from learning about” these realities (Macatè v. Lithuania, 61435/19, p. 213) or arbitrarily limiting information of a sexual nature. The creation and divulgation of knowledge in SRH matters needs to be evidence- and human-rights-based, produced through documentation of diverse experiences, and inclusive of all people [8; 9]. In sensitive matters such as public discussion of sex education for the young audience, the authorities must be guaranteed access based on “the criteria of objectivity, pluralism, scientific accuracy and usefulness” (Bayev and Others v. Russia, 67667/09, p. 29). With regard to the judgments here analysed, it is worth noting that the interpretations of the Convention in 2023, 2017, 2012 and 1976 have shifted in the argumentation made.

### Gender reassignment surgery

The association between sexual behaviour stigma, mental health issues and impaired healthcare access in both low- and high-income countries has been widely demonstrated in the literature [45–47], with an increased risk of depression, anxiety, suicidality and self-harm in transgender and gender diverse individuals (TGD) [48]. In particular, recent studies have drawn attention to the limited access to healthcare that transgender and gender diverse individuals experience in the EU ([14]:196–201; [49–51]). These findings emphasise the necessity for amendments to existing stigmatising legislation and social acceptance of gender affirming care [52].

Over the last two decades a range of interdisciplinary models of gender-affirming care have been evaluated within healthcare settings, e.g. in behavioural therapy, with a view to helping TGD youth navigating gender transition and reducing gender dysphoria [48, 53–56]. These clinical practices include non-directive counselling and are aimed to prioritise mental health by fostering emotional and psychosomatic wellbeing [48].

A focus on evidence-based intervention approaches and future qualitative research studies is thus imperative (1) to improve life and reproductive health experiences of transgender and gender diverse individuals during and post medical transition [54, 57]; and (2) to educate health providers to the needs of TGD patients and related additional barriers to accessing gender-affirming, such as inadequate support from family members or in a clinical setting [57], insurance coverage, the remoteness of a gender clinic, belonging to minority groups [48, 54, 55]. Moreover, further discussion about gender-affirming care access is essential to better inform health providers and TGD patients about (a) the risks and possibilities of gender reassignment surgeries, (b) collateral gynaecological conditions and non-invasive gynaecological treatment options [56], such as contraception for transmasculine patients or stopping testosterone for transmasculine individuals who wish to carry a pregnancy [55, 57], as well as (c) the long-term effects of hormone therapy, such as cardiovascular risks [58], musculoskeletal conditions, fertility issues. The consistent implementation of these measures respects all the four principles of medical ethics, i.e. “autonomy”, “beneficence”, “non-maleficence” and “justice” [15, 59]. Therefore, the overarching objective is the attainment of healthcare standards for transgender and diverse patients by activating gender-inclusive resources related to sexual and reproductive health, see e.g. the World Professional Association of Transgender Health (WPATH) Standards of Care (SOC) [54, 55, 58].

## Conclusions

This paper presented an ethical analysis of judgments handed down by the Court of Human Rights against Member States that are parties to the European Convention on Human Rights. Our analysis highlighted a number of shortcomings in access to SRH services in the EU from 1976 to 2023. It showed that to properly address the discourse on access to abortion or the issue of self-determination over family planning or childbirth, access to SRH must be understood as a human right for all women and TGD who wish to carry a pregnancy, in particular for racialised and marginalised individuals ([14]:129). This comprises the right to “sexual autonomy” and “reproductive dignity” in healthy settings ([14]:56, 65), including safe abortion, reproductive assisted technologies, home birth, “gender freedom” and sex education ([8]; 9; [14]: 65, 162, 188). These rights align with the four principles of bioethics and the foundational framework of reproductive justice, such as the right to have children, not to have children and to parent them in safe environments ([14]:168–174). Violations of SRH services are indicators of social and public health policies that undermine individual empowerment, and thus increase the vulnerability of marginalised groups. This emphasises the necessity to integrate culturally grounded and justice-oriented approaches, when it comes to conducting root cause analysis, implementing corrective actions and preventing violations of SRH in a given social, economic and political context ([14]: 70,111–113, 128, 162). Indeed, the analysed ECtHR judgments affected the lives and narrative of individuals whose sexual and reproductive choices were medicalised into a morbidity or stigmatised as a violation of European national moralities. It is therefore imperative to regularly monitor the different national European policies on access to informed reproductive self-determination in accordance with the Convention and to establish accountable mitigation measures. This is necessary to (1) shift the discourse from reproductive choices, which are unavailable to marginalised people, to reproductive rights, and (2) secure equitable and non-discriminatory access to SRH in response to the evolving reproductive health needs of all people and the complexity of scientific advances in biomedicine.

### Limitations

Methodologically, the authors acknowledge that the list of cases presented herein may not be exhaustive, as it reflects the judgements included in the HUDOC database. Furthermore, the authors intentionally searched the HUDOC database for violations of the right to informed reproductive self-determination and the right to freedom of information in matters of sex education. We applied reproductive “abuse”, “coercion” and “violence” as synonyms for reproductive control [59–61]. The search was restricted to the objectives established by the European Parliament’s plenary session in June 2021. These aimed to guarantee access to “legal and safe abortion”, eliminate obstacles to sexual and reproductive health rights services and education, and eradicate any “form of violence against women and girls”: –https://www.europarl.europa.eu/news/en/press-room/20210621IPR06637/eu-countries-should-ensure-universal-access-to-sexual-and-reproductive-health. The search was therefore not extended to other areas of sexuality.

## Data Availability

No datasets were generated or analysed during the current study.

## Abbreviations

SRH: Sexual and reproductive health

## References

[1] De Zordo S, Mishtal J, Zanini G, Gerdts C. `The First Difficulty is Time´: the impact of gestational age limits on reproductive health and justice in the context of cross-border travel for abortion care in Europe. Soc Sci Med. 2023;321:115760. 10.1016/j.socscimed.2023.115760. 36801749 10.1016/j.socscimed.2023.115760

[2] Wigand M, Orzechowski M, Nowak M, Becker T, Steger F. Schizophrenia, human rights and access to health care: a systematic search and review of judgments by the European court of human rights. Int J Soc Psychiatry. 2021;67(2):168–74. 32674633 10.1177/0020764020942797

[3] Orzechowski M, Wigand E, Nowak M, Becker T, Steger F. Post-traumatic stress disorder, human rights and access to healthcare: an analysis of judgments of the European Court of Human Rights from an ethical perspective. Eur J Psychotraumatol. 2021;12(1):1930704. 10.1080/20008198.2021.1930704. 34211639 10.1080/20008198.2021.1930704 PMC8221123

[4] Skuban-Eiseler T, Orzechowski M, Steger F. Access to healthcare for disabled individuals: an analysis of judgments of the European court of human rights from an ethical perspective. Front Public Health. 2023a;10:1015401. 10.3389/fpubh.2022.1015401. 36703847 10.3389/fpubh.2022.1015401 PMC9871461

[5] Skuban-Eiseler T, Orzechowski M, Steger F. Access to healthcare for people living with HIV: an analysis of judgments of the European court of human rights from an ethical perspective. Front Public Health. 2023b;11:1193236. 10.3389/fpubh.2023.1193236. 37377554 10.3389/fpubh.2023.1193236 PMC10292927

[6] Skuban-Eiseler T, Orzechowski M, Steger F. Restriction of access to healthcare and discrimination of individuals of sexual and gender minority: an analysis of judgments of the European court of human rights from an ethical perspective. Int J Environ Res Public Health. 2020;19:2650. 10.3390/ijerph19052650. 10.3390/ijerph19052650 PMC8909593 35270340

[7] Tietze F-A, Orzechowski M, Nowak M, Steger F. Access to healthcare for minors: an ethical analysis of judgments of the European Court of Human Rights. Healthcare. 2021;9:1361. 10.3390/healthcare9101361. 34683040 10.3390/healthcare9101361 PMC8544556

[8] Subha Sri B. 2022. Rights-based knowledge creation in sexual and reproductive health: an introductory guide. Sex Reproductive Health Matters https://www.srhm.org/rights-based-knowledge-creation/

[9] Committeeon Economic, Social and Cultural Rights. CESSCR general comment no. 14 on the highest attainable standard of health. 2000. E/C. 12/2000/4. https://www.ohchr.org/sites/default/files/Documents/Issues/Women/WRGS/Health/GC14.pdf.

[10] Braun V, Clarke V. Using thematic analysis in psychology. Qual Res Psychol. 2008;3(2):77–101.

[11] Chor J, Watson K. Reproductive ethics in clinical practice: preventing, initiating, managing pregnancy and delivery. essays inspired by the Maclean center for clinical medical ethics lecture series. Oxford: 2021.

[12] Gilliam M, Roberts D. 2021. Why Reproductive Justice Matters in Reproductive Ethics. In J. Chor, and K. Watson, Reproductive Ethics in Clinical Practice. Preventing, Initiating, Managing Pregnancy and Delivery. Oxford: 17–28.

[13] Crenshaw K. Mapping the margins: intersectionality, identity politics, and violence against women of color. Stanford Law review. 1991;43(6):1241–99.

[14] Ross LJ, Solinger R. Reproductive justice: an introduction (Vol. 1). Series: Reproductive justice: a new vision for the 21st century. Oakland, CA: 2017.

[15] Beauchamp TL, Childress JF. Principles of biomedical ethics. 8th ed. Oxford, UK: Oxford University Press; 2019.

[16] Braun V, Clarke V. Reflecting On Refexive Thematic Analysis. Qual Res Sport Exerc Health. 2019;11(4):589–97.

[17] Starr KJ. 5 Facts About Catholics in Europe. example%2 C%20at%20least%20three,75%25%20of%20Lithuanians%20are%20Catholic; 2018. https://www.pewresearch.org/short-reads/2018/12/19/5-facts-about-catholics-in-europe/#:~:text=For%20. Access 29 February 2024.

[18] Freedman L, Stulberg D. 2021. Religiously Affiliated Healthcare Institutions: An Ethical Analysis of What They Mean for Patients, Clinicians and Our Health System. In J. Chor, and K. Watson, Reproductive Ethics in Clinical Practice. Preventing, Initiating, Managing Pregnancy and Delivery. Oxford: 29–43.

[19] Stulberg DB, Jackson RA, Freedman LR. Referrals for Services Prohibited In Catholic Health Care Facilities. Perspect Sex Reprod Health. 2016;48(3):111–7. 27467888 10.1363/48e10216

[20] Chervenak F, McCullough LB. 2021. Professional Ethics in Obstetric Practice, Innovation and research. In J. Chor, and K. Watson, Reproductive Ethics in Clinical Practice. Preventing, Initiating, Managing Pregnancy and Delivery. Oxford: 197–211.

[21] Grimes L, O’ Shaughnessy A, Roth R, Carnegie A, Duffy DN. Analysing my options: experiences of Ireland’s abortion information and support service. BMJ Sex Reprod Health. 2022;48(3):222–6. 10.1136/bmjsrh-2021-201424. 35288458 10.1136/bmjsrh-2021-201424

[22] Watson K. Abortion as a moral good. Lancet. 2019;393(March 23):1196–97. 30910296 10.1016/S0140-6736(19)30581-1

[23] Gong D, Liu Y-L, Zheng Z, Tian Y-F, Li Z. An overview on ethical issues about sperm donation. Asian J Androl. 2009;11(6):645–52. 10.1038/aja.2009.61. 19767762 10.1038/aja.2009.61 PMC3735320

[24] Negro F, Beck R, Cotoia A, Varone MC. Posthumous sperm retrieval: a procreative revolution. Med Glas (Zenica). 2021;18(1):114–21. 10.17392/1256-21. 33219640 10.17392/1256-21

[25] Jones GL, Folan A-M, Phillips B, Anderson RA, Ives J. Reproduction in life and death: should cancer patients with a poor prognosis be offered fertility preservation interventions? Reproduction & Fertility. 2023;4(4):e230047. 37869895 10.1530/RAF-23-0047 PMC10692684

[26] Polyakov A, Rozen G. Exploring the complexities of posthumous reproduction in fertility preservation for oncology patients with poor prognosis. Reprod Fertil. 2023;4(4):e230072. 10.1530/RAF-23-0072. 37962498 10.1530/RAF-23-0072 PMC10762574

[27] Steering Committee on Bioethics. CDBI-CO-GT3. 2003;13. https://www.coe.int/t/dg3/healthbioethic/activities/04_human_embryo_and_foetus_en/CDBI-CO-GT3(2003)13E.pdf. Access 29 February 2024.

[28] Bayefsky MJ. Comparative preimplantation genetic diagnosis policy in Europe and the USA and its implications for reproductive tourism. Reproductive Biomedicine & Society Online. 2016;3:41–7. 10.1016/j.rbms.2017.01.001. 28959787 10.1016/j.rbms.2017.01.001 PMC5612618

[29] Piersanti V, Consalvo F, Signore F, Del rio A, Zaami S. Surrogacy and “procreative tourism”. What does the future hold from the ethical and legal perspectives? Medicina (Kaunas). 2021;57(1):47. 10.3390/medicina57010047. 33429930 10.3390/medicina57010047 PMC7827900

[30] Binet J-R. Comparative study on access of persons conceived by gamete donation to information on their origin. Council Europe; 2022. pp. 1–40. Access 29 Feb 2024.

[31] Metzler I. Nationalizing embryos’: the politics of human embryonic stem cell research in Italy. BioSocieties. 2007;2(4):413–27. 10.1017/S1745855207005856.

[32] Isasi R, Ginoza M, Jongsma K, Assen L, Fabbri M. Mending the gaps: ethically sensitive cells and the evolution of European stem cell policy. Regen Med. 2022. 10.2217/rme-2022-0043. 35670098 10.2217/rme-2022-0043

[33] Jadva V, Imrie S. Embryo donation: motivations, experiences, parenting, and child adjustment. Fertil Steril. 2023;119(1):11–4. 10.1016/j.fertnstert.2022.09.012. 36396495 10.1016/j.fertnstert.2022.09.012

[34] Zimon AE, Shepard DS, Prottas J, Rooney KL, Ungerleider J, Halasa-Rappel YA, et al. Embryo donation: survey of *in-vitro* fertilization (IVF) patients and randomized trial of complimentary counseling. PLoS One. 2019;14(8):e0221149. 10.1371/journal.pone.0221149. 31415660 10.1371/journal.pone.0221149 PMC6695140

[35] Leach Scully J, Haimes E, Mitzkat A, Porz R, Rehmann-Sutter C. Donating embryos to stem cell research. The problem of gratitude. J Bioeth Inq. 2012;9(1):9–28. 10.1007/s11673-011-9352-9 PMC3714553 23180197

[36] Montanari Vergallo G. „Freedom of scientific research and embryo protection under Italian and European court of human rights’ Jurisprudence. Brief European legislation overview. Eur J Health Law. 2021;28(1):3–25. 10.1163/15718093-BJA10036. 33652382 10.1163/15718093-BJA10036

[37] Grünebaum A, McCullough LB, Brent RL, Arabin B, Levene MI, Chervenak FA. Perinatal risks of planned home birth in the united States. Am J Obstet Gynecol. 2015;212:el350–6. 10.1016/j.ajog.2014.10.021 25446661

[38] Wax JR, Lucas FL, Lamont M, Pinette MG, Cartin A, Blackstone J. Maternal and newborn outcomes in planned home birth vs planned hospital births: a meta-analysis. Am J Obstet Gynecol. 2010;203:e2431-8. 10.1016/j.ajog.2010.05.028 20598284

[39] Gyte G, Newburn M, Macfarlane A. 2010. Critique of a Meta-analysis by wax and colleagues which has claimed that there is a three-times greater risk of neonatal death among babies without congenital anomalies planned to be born at home. National Childbirth Trust. http://fr.scribd.com/doc/34065092/Critique-of-a-meta-analysis-by-Wax. Access 29 February 2024.

[40] Quattrocchi P. Policies and practices on out-of-hospital birth: a review of qualitative studies in the time of Coronavirus. Curr Sex Health Rep. 2023;15(1):36–48. 10.1007/s11930-022-00354-7. 36530373 10.1007/s11930-022-00354-7 PMC9735103

[41] KóczéA. Gender, ethnicity and class: romani women’s political activism and social struggles. Dissertation by Ángela Kóczé submitted to the central European university, department of sociology and social anthropology. 2011. https://www.etd.ceu.edu/2012/sphkoc01.pdf.

[42] Hanschmidt F, Linde K, Hilbert A, Riedel-Heller S, Kersting A. 2016. „Abortion Stigma: A Systematic Review. Perspectives on Sexual and Reproductive Health, Vol. 48: 4 (December 2016): 169–177. 10.1363/48e8516 27037848

[43] Jim A, Magwentshu M, Mnezel J, Küng SA, August S-A, van Rooyen J, Chingwende R, Pearson E. Stigma towards women requesting abortion and association with health facility staff facilitation and obstruction of abortion care in South Africa. Front Glob Womens Health. 2023;15 June 2023 Sec Contraception and Family Planning 4. 10.3389/fgwh.2023.1142638. 10.3389/fgwh.2023.1142638 PMC10311091 37396569

[44] Kumar A, Hessini L, Mitchell EMH. Conceptualising abortion stigma. Cult Health Sex. 2009;11(6):625–39. 19437175 10.1080/13691050902842741

[45] Hart G, Wellings K. Sexual behaviour and its medicalisation: in sickness and in health. BMJ. 2002;324(7342):896–900. 10.1136/bmj.324.7342.896. 11950742 10.1136/bmj.324.7342.896 PMC1122837

[46] Dourado I, Crosland Guimarães MD, Nogueira Damacena G, Magno L, de Borges Souza Júnior PR, Landmann Szwarcwald C, et al. Sex work stigma and non-disclosure to health care providers: data from a large RDS study among FSW in Brazil. BMC Int Health Hum Rights. 2019;5(1):8. 10.1186/s12914-019-0193-7. 10.1186/s12914-019-0193-7 PMC6399834 30832659

[47] Daniels I, Anthony T, Peavie J, Miesfled N, Pyatt T, Robinson D, et al. Black men who have sex with men with HIV and providers in HIV care settings reflect on stigma reducing strategies to promote engagement in health care. AIDS Patient Care STDS. 2022;36(S1):28–35. 10.1089/apc.2022.0102. 10.1089/apc.2022.0102 36178381

[48] Huit TZ, Coyne C, Chen D. State of the science: gender-affirming care for transgender and gender diverse youth. Behav Ther. 2024;55:1335–47. 39443069 10.1016/j.beth.2024.02.010

[49] Skuban-Eiseler T, Orzechowski M, Steger F. Why do transgender individuals experience discrimination in healthcare and thereby limited access to healthcare? An interview study exploring the perspective of German transgender individuals. Int J Equity Health. 2023;(1):211. 10.1186/s12939-023-02023-0. 37817187 10.1186/s12939-023-02023-0 PMC10566060

[50] Winter S, Diamond M, Green J, Karasic D, Reed T, Whittle S, Wylie K. Transgender people: health at the margins of society. Lancet. 2016;23(10042):390–400. 10.1016/S0140-6736(16)00683-8. 10.1016/S0140-6736(16)00683-8 27323925

[51] Kiely E, Millet N, Baron A, Kreukels BPC, Doyle DM. Unequal geographies of gender-affirming care: a comparative typology of trans-specific healthcare systems across Europe. Soc Sci Med. 2024;356:117145. 39067377 10.1016/j.socscimed.2024.117145

[52] Falck F, Bränström R. The significance of structural stigma towards transgender people in health care encounters across Europe: health care access, gender identity disclosure, and discrimination in health care as a function of national legislation and public attitudes. BMC Public Health. 2023;23:1031. 37259082 10.1186/s12889-023-15856-9 PMC10230714

[53] Coyne CA, Yuodsnukis BT, Chen D. Gender dysphoria: optimizing healthcare for transgender and gender diverse youth with a multidisciplinary approach. Neuropsychiatr Dis Treat. 2023;19:479–93. 10.2147/ndt.S359979. 36879947 10.2147/NDT.S359979 PMC9985385

[54] Comer JS. State of the science in behavior therapy: taking stock and looking forward. Behav Ther. 2024;55(6):1101–13. 39443055 10.1016/j.beth.2024.07.007

[55] Simko S, Popa O, Stuparich M. Gender affirming care for the minimally invasive gynecology surgeon. Curr Opin Obstet Gynecol. 2024;36:301–12. 38597457 10.1097/GCO.0000000000000956

[56] Defant MJ. Reevaluating gender-affirming care: biological foundations, ethical dilemmas, and the complexities of gender dysphoria. Marital Therapy. 2025;51(2):200–10. 10.1080/0092623X.2025.2456066. 10.1080/0092623X.2025.2456066 39841090

[57] Schechner J, Zayhowski K, Haghighat D, Ruderman M. Transgender and gender diverse patients‘ experiences with pregnancy-related genetic discussions: a qualitative study. Journal of Genetic Counselors. 2024;34:e2018. 10.1002/jgc4.2018. 10.1002/jgc4.2018 PMC11923581 40111221

[58] Dolendo I, Zhao L, Bluebond-Langner R, Anger JT. Bridging the evidence gap in gender-affirming care: urgent research needs. BJU International 2025 Jun. 2025. 10.1111/bju.16783. 2Online ahead of print. 10.1111/bju.16783 40457589

[59] Stugart LK, Larson SC, Lipsey KL, Owens G. Gender-affirming care is not standard care in occupational therapy: a scoping review. Am J Occup Ther. 2025;79(2):790280060. 10.5014/ajot.2025.050883 39976641

[60] Link BG, Phelan JC. Stigma and its public health implications. Lancet. 2006;367(9509):528–9. 10.1016/S0140-6736(06)68184-1. 16473129 10.1016/S0140-6736(06)68184-1

[61] Tarzia L, Hegarty K. A conceptual re-evaluation of reproductive coercion: centring intent, fear and control. Reprod Health. 2021;18:87. 10.1186/s12978-021-01143-6. 33906687 10.1186/s12978-021-01143-6 PMC8077849

